# Effect of Osteoporosis Treatments on Osteoarthritis Progression in Postmenopausal Women: A Review of the Literature

**DOI:** 10.1007/s11926-024-01139-8

**Published:** 2024-02-19

**Authors:** Wang-Chun Ho, Chung-Chih Chang, Wen-Tien Wu, Ru-Ping Lee, Ting-Kuo Yao, Cheng-Huan Peng, Kuang-Ting Yeh

**Affiliations:** 1 School of Medicine, Tzu Chi University, Hualien, Taiwan; 2 Department of Orthopedics, Hualien Tzu Chi Hospital, Buddhist Tzu Chi Medical Foundation, Hualien, Taiwan; 3 Institute of Medical Sciences, Tzu Chi University, Hualien, Taiwan; 4 Graduate Institute of Clinical Pharmacy, Tzu Chi University, Hualien, Taiwan

**Keywords:** Osteoporosis, Osteoarthritis (OA), Bisphosphonate, Selective estrogen receptor modulators (SERMs), Parathyroid hormone (PTH), Denosumab

## Abstract

**Purpose of Review:**

The purpose of this literature review was to determine if medications used to treat osteoporosis are also effective for treating osteoarthritis (OA).

**Recent Findings:**

A total of 40 relevant articles were identified. Studies were categorized into those (1) discussing estrogen and selective estrogen receptor modulators (SERMs), (2) bisphosphonates, (3) parathyroid hormone (PTH) analogs, and (4) denosumab, and (5) prior review articles. A large amount of evidence suggests that estrogen and SERMs are effective at reducing OA symptoms and disease progression. Evidence suggests that bisphosphonates, the most common medications used to treat osteoporosis, can reduce OA symptoms and disease progression. In vivo studies suggest that PTH analogs may improve the cartilage destruction associated with OA; however, few human trials have examined its use for OA. Denosumab is approved to treat osteoporosis, bone metastases, and certain types of breast cancer, but little study has been done with respect to its effect on OA.

**Summary:**

The current evidence indicates that medications used to treat osteoporosis are also effective for treating OA. Estrogen, SERMs, and bisphosphonates have the most potential as OA therapies. Less is known regarding the effectiveness of PTH analogs and denosumab in OA, and more research is needed.

## Introduction

Osteoporosis (OP) and osteoarthritis (OA) are two important skeletal disorders primarily associated with aging [1, 2, 3•, 4]. OP is a condition characterized by a loss of bone mineral density (BMD), resulting in bones with decreased structural integrity making them more prone to fractures [1, 2, 3•, 4]. On the other hand, OA is a complex inflammatory disease affecting various joint tissues [5, 6]. While cartilage degeneration is a hallmark feature of end-stage disease, OA disrupts joint homeostasis through chronic inflammation, impacting the synovium, subchondral bone, ligaments, and periarticular structures. In advanced stages, loss of cartilage quality and quantity leads to bone-on-bone contact, reducing joint mobility and function, causing severe pain [5, 6]. Interestingly, some studies have reported that higher BMD was associated with a reduced risk of OA in the knee [3•, 7]. Thus, OP and OA may be intersecting in terms of BMD.

The primary medications used to treat OP include bisphosphonates, estrogen, and selective estrogen receptor modulators (SERMs) [3•]. Bisphosphonates bind with high affinity to the mineral matrix of bone and inhibit osteoclast resorption of the bone, leading to a decrease in bone turnover and a net gain in bone mass [1, 3•, 4]. Estrogen affects bone through via a number of mechanisms [1, 3•, 4]. Estrogen can (1) lower the sensitivity of bone to the effects of parathyroid hormone (PTH), and thus reduce bone resorption; (2) increase the production of calcitonin, and thus inhibit bone resorption; (3) accelerate calcium resorption by the intestine, and thus increase serum calcium concentration which promotes increase of bone mass; (4) reduce calcium excretion from the kidneys, and subsequently increases serum concentration; and (5) directly affect bone turnover due to the presence of estrogen receptors in bone. Other less common osteoporosis treatments, such as testosterone, calcitonin, and PTH analogs, operate through mechanisms distinct from bisphosphonates [3•, 8]. Testosterone stimulates osteoblast-dependent bone formation more than osteoclast-dependent bone resorption, contributing to a positive skeletal balance. Calcitonin regulates calcium levels and inhibits osteoclast activity, impacting bone remodeling. PTH analogs, like teriparatide, directly stimulate osteoblastic formation, offering an anabolic approach to enhance bone density [8].

The purpose of this report is to review the literature and examine the effect of commonly used anti-osteoporotic medications on the development and progression of OA in postmenopausal women. The findings of this review may assist clinicians in treating aging women with concomitant osteoporosis and OA.

## Method

### Search Strategy and Inclusion Criteria

A systematic search was conducted using the PubMed database that included the year 2013 through May 2023. The design and development of this study, and the database search and reporting followed the Preferred Reporting Items for Systematic Reviews and Meta-Analyses (PRISMA) 2020 guidelines (http://www.prisma-statement.org/).

For the database search, the following keyword combinations were used: “postmenopausal AND osteoarthritis OR OA” and “ postmenopausal AND osteoporosis treatment OR anti-resorptive medication”; “estrogen OR hormone replacement therapy OR estrogen-related medication”; “selective estrogen-receptor modulators OR SERMs OR raloxifene OR tamoxifen OR levormeloxifene”; “ bisphosphonate OR alendronate OR zoledronic acid OR risedronate OR tiludronate OR pamidronate OR ibandronate OR Zometa OR Reclast OR Fosamax OR Actonel OR Boniva”; “parathyroid hormone OR PTH-related peptide OR Tymlos OR teriparatide OR abaloparatide OR Forteo”; and “osteoarthritis AND denosumab OR Prolia OR Romosozumab.”

Reference lists of all included articles were also screened for other potentially relevant articles that should be included in this review. Studies eligible for inclusion were those published in English and focused on postmenopausal women. Each article identified was independently reviewed and included in this review if it was a human or animal study investigating the effect of osteoporosis medications on OA. In addition, prior literature reviews and systematic review and meta-analyses relevant to the effect of medications used to treat osteoporosis on OA were also included.

### Data Extraction

Each included study was read closely, and data/information extracted included (1) whether it was an animal or human study; (2) what animal model was used (for animal studies); (3) basic patient information (age and sex) and the number of patients (for human studies); (4) study design (e.g., prospective, retrospective, literature review/analysis (for human studies)); (5) the joint(s) studied; (6) the cell type studied; (7) the drug studied; (8) drug route of administration, dosage, frequency, and treatment length; (9) methods of outcome assessment before and after treatment; (10) results/outcomes; and (11) association between treatment and results/outcomes.

## Results

The literature search using the abovementioned criteria identified 40 articles relevant to the topic. The studies included in this article have been categorized into those discussing (1) estrogen and SERMs, (2) bisphosphonates, (3) parathyroid hormone, (4) denosumab, and (5) prior reviews.

### Estrogen and Selective Estrogen Receptor Modulators (SERMs)

In postmenopausal women, the decrease of estrogen level is a causative factor in loss of BMD and is associated with a subsequent increased risk of fractures [1, 3•, 4]. Hormone therapy is now a standard treatment for osteoporosis. Hormones, especially estrogen and SERMs, are used for preventing osteoporosis.

#### Estrogen: Human Studies

Cheng et al. [9•] provided an overview of osteoporosis due to hormone imbalance. While use of estrogen by postmenopausal women can increase the risk of endometrial cancer, it is effective in treating estrogen-deficiency osteoporosis [9•]. While detailed discussion of the mechanisms of action of estrogen with respect to osteoporosis treatment is beyond the scope of this review, estrogen binds to estrogen receptors and subsequently inhibits osteoclast formation and bone resorptive activity.

In a very recent study, Yang et al. [10] performed a meta-analysis to examine the effects of estrogen on OA. The analysis included 11 studies with approximately 12,000 participants, with about half receiving estrogen to treat OA and the other half receiving treatments other than estrogen. Notably, patients treated with estrogen had a significantly lower collagen cross-linked C-telopeptide type 1 (CTX-I) level and a significantly higher bone Gla protein (BGP) level. Importantly, there were fewer patients treated with estrogen with joint pain than those treated with other medications, and those treated with estrogen with joint pain had lower subjective pain scores than patients treated with other therapies. However, the interpretation of outcomes of that meta-analysis might be limited due to a lack of strict criteria for inclusion, particularly in not explicitly incorporating studies utilizing the gold standard OA definition.

#### Estrogen: Animal Studies

Studies using animal models of a postmenopausal state and decreased estrogen level have indicated that maintaining a normal physiologic estrogen level can reduce OA symptoms [11–13]. Xu et el. [12] used a murine model of a postmenopausal state (ovariectomy mice) to study the effects of estrogen deficiency on cartilage and subchondral bone remodeling. The overall findings were that estrogen deficiency resulted in resorption of subchondral bone and degeneration of articular cartilage.

Corciulo et al. [11] also used a murine model of a postmenopausal state (ovariectomy mice) and found that treatment with estrogen prevented damage and thickening of the synovium, protected against loss of subchondral trabecular bone, and improved motor activity and reduced pain sensitivity. Psoralen is an estrogen analog that is derived from plants. Huang et al. [13] studied the effects of psoralen in a rabbit OA model that was created by transection of the anterior cruciate ligament. The results showed that psoralen decreased the destruction of cartilage tissue reflected by Osteoarthritis Research Society International (OARSI) score and increased subchondral BMD, trabecular thickness and number.

#### SERMs

Selective estrogen receptor modulators (SERMs), also referred to estrogen receptor agonist/antagonists (ERAAs), are a class of drugs that act on estrogen receptors (ERs) [14•]. Unlike pure ER agonists and antagonists, the effects of SERMs are different in different tissues, and thus can inhibit or stimulate ERs in different tissues [14•, 15]. SERMs are used to treat a wide variety of conditions, including ovulatory dysfunction in the management of infertility, treatment and prevention of postmenopausal osteoporosis, treatment and reduction in risk of breast cancer, and treatment of dyspareunia and other symptoms of menopause [15].

A number of studies and review articles have examined the effect of different SERMs on OA [15–19]. Raloxifene is a second-generation SERM that has an estrogen-agonistic effect on bone; it increases BMD and bone mass by decreasing bone resorption [14•]. It is indicated in the treatment and prevention of postmenopausal osteoporosis and for risk reduction of invasive breast cancer in postmenopausal women [14•].

Xiao et al. [19] reviewed the use of “estrogen-related” drugs for the treatment of OA and concluded that although there are some inconsistencies in the literature, SERMs (and estrogen) may be particularly effective in lowering OA incidence and providing relief from OA-related pain, especially for individuals with early-stage OA or those with OA related to osteoporosis (reduced BMD related to high remodeling in subchondral bone). In a prior review of the literature (2014), Lugo et al. [15] also concluded that SERMs may be particularly effective in postmenopausal women with osteoporotic OA (reduced BMD related to high remodeling in subchondral bone).

Bei et al. [17] showed that raloxifene slows cartilage degradation and improves subchondral bone micro-architecture in a rat postmenopausal model (ovariectomized) with patellofemoral joint OA. An in vitro model study showed that raloxifene reversed OA-like alterations in rat chondrocytes [18]. Specifically, that study sheds light on the potential of raloxifene to influence and mitigate OA-related molecular alterations, presenting a multi-faceted perspective on its impact at the genetic, epigenetic, and proteomic levels.

Studies reporting effects of estrogen and SERMs in OA are summarized in Table 1.

**Table 1:** Studies reporting effects of estrogen and SERMs in OA

Author’s name	Year	Type	Drug/therapy	Effects in OA
Corciulo C et al. [11]	2022	Animal	Estrogen	Prevented damage and thickening of the synovium, protected against loss of subchondral trabecular bone, improved motor activity, and reduced pain sensitivity
Huang K et al. [13]	2022	Animal	Psoralen (estrogen analog)	Decreased cartilage tissue destruction, increased subchondral BMD, trabecular thickness and number, and decreased Osteoarthritis Research Society International (OARSI) scores
Lugo L et al. [15]	2014	Review	Estrogen therapy	Reduction of cartilage damage in osteoporosis-related OA
Bei MJ et al. [17]	2020	Human	Raloxifene	Improved subchondral bone micro-architecture via modulating cartilage metabolism by decreasing MMP-13, ADAMTS-4, and caspase-3 and increasing Col-II
Kavas A et al. [18]	2013	Animal	Raloxifene	Reversed OA-like alterations in rat chondrocytes via modulating OA-related genes, apoptosis, MMP-13 and MMP-3 protein expressions, and increasing aggrecan and Col-II
Xiao YP et al. [19]	2016	Review	Estrogen-related drugs and therapy	Reduction of cartilage damage in osteoporosis-related OA
Xu X et al. [12]	2019	Review	Estrogen	Modulating cartilage and subchondral bone remodeling
Yang X et al. [10]	2023	Meta-analysis	Estrogen	Summarize clinical status and protective effects

Abbreviations: *BMD*, bone mineral density; *OA*, osteoarthritis; *SERMs*, selective estrogen receptor modulators; *MMP-3*, matrix metalloproteinase-3; *MMP-13*, matrix metalloproteinase-13; *ADAMTS-4*, ADAM metallopeptidase with thrombospondin type 1 motif 4; *Col-II*, collagen type II

### Bisphosphonates

Bisphosphonates prevent the loss of bone density and are the most commonly used drugs to treat osteoporosis [20]. Bone tissue is always undergoing remodeling, and the balance of bone tissue is maintained by osteoblasts creating bone and osteoclasts catalyzing bone [20]. Bisphosphonates work by causing osteoclasts to undergo apoptosis, and thus slow bone loss [20].

A large body of evidence shows that bisphosphonates reduce the risk of fracture in postmenopausal women with osteoporosis [20]. Bisphosphonates are also used to treat other conditions such as Paget’s disease, bone metastasis, multiple myeloma, and primary hyperparathyroidism [20]. Alendronate was the first bisphosphonate approved by the United States Food and Drug Administration (US FDA) as a treatment of osteoporosis in 1995, and many others have been approved and found wide use since then.

Although bisphosphonates are not generally used for the treatment of OA, a number of studies have indicated that they can reduce cartilage damage and symptoms of OA [21–28].

#### Prior Reviews

Our review of the literature identified 4 prior reviews examining the use of bisphosphonates with respect to OA published from 2013 to 2021 [22, 26–28]. An in-depth analysis of each review is beyond the scope of this article; rather, the primary findings are summarized. The earliest review was published in 2013 by Davis et al. [27]. The systematic review and meta-analysis included 13 studies with 3832 participants. The authors concluded that there was limited evidence that bisphosphonates are effective for treating OA pain. However, they acknowledged there was marked heterogeneity in the included studies and a lack of long-term follow-up data.

Vaysbrot et al. [28] performed a systematic review and meta-analysis of randomized controlled trials to examine if bisphosphonates are effective in treating knee OA. The analysis included 7 trials with approximately 3000 patients. Most patients were treated with oral risedronate (about 2800). Overall there were no significant differences in pain, functional outcomes, or radiographic progression between patients who received a drug treatment and those that received placebo. However, based on the analysis, the authors concluded that bisphosphonates may be useful in subsets of patients who have a high rate of subchondral bone turnover.

Two reviews were published in 2021. The first by Fernández-Martín et al. [26] focused on preclinical animal studies published from 2000 to 2020. Overall, the authors concluded that bisphosphonates appeared to reduce osteoarthritic changes in a dose dependent manner, and that earlier initiation of treatment may produce better results. However, the authors indicated the heterogeneity of the studies and lack of long-term follow-up prevented definitive conclusions. The second review published in 2021 by Eriksen et al. [22] included animal and human studies and concluded that although in vitro and animal studies suggest that bisphosphonates may be of value in the treatment of OA, evidence from human studies is lacking and this may be due to study heterogeneity and lack of long-term follow-up.

#### Animal Studies

Our review identified 1 animal study meeting the search criteria. She et al. [21] studied the effect of zoledronic acid, a third-generation bisphosphonate, using a rabbit model of knee OA. The results showed that zoledronic acid increased subchondral bone density, reduced the degeneration of articular cartilage, and exerted a chondroprotective effect in a dose-dependent manner.

#### Human Studies

Three human studies published from 2017 to 2022 examining bisphosphonates and OA were identified in our review. Fu et al. [23] performed a national cohort study in Taiwan of persons with osteoporosis and newly diagnosed OA, and the risk of undergoing total knee arthroplasty (TKA). The study included about 16,000 bisphosphonate users and 124,000 persons not treated with any anti-osteoporosis drug seen from 2009 to 2012. The results showed that patients treated with a bisphosphonate had a significantly reduced risk of undergoing TKA (adjusted hazard ratio [HR] = 0.76, 95% confidence interval [CI]: 0.69 to 0.83, *p* < 0.001). Adherence to treatment and longer treatment duration were also associated with reduced risk, and bisphosphonate users used significantly less pain medication than nonusers.

Hayes et al. [24] used data from the Osteoarthritis Initiative (OAI) to determine if bisphosphonate use protected against 2-year radiographic progression of knee OA. The study focused on women aged 50 and older with varying degrees of baseline radiographic knee OA, excluding individuals with severe OA, knee prosthetics, or self-reported exposure to non-bisphosphonate osteoporosis medications. While the sample size was relatively small (approximately 2000 women of whom 346 used bisphosphonates), the authors concluded that there may be a protective effect in patients with early-stage OA. The effect was specifically notable in patients who were not overweight, and the medications were less effective in patients with more advanced disease and those with more weight-bearing joint stress.

In an interesting, recent study, Kawai et al. [25] retrospectively reviewed the records and standing whole leg radiographs of patients (*N* = 398) who received a TKA. The authors measured the joint space narrowing in the non-arthritic hip and found that the rate of narrowing was significantly less in patients who were taking bisphosphonates than those who were not. Despite these evidences, there are currently no available human data on the relative efficacy for the prevention or against the progression of OA between specific bisphosphonates.

Studies reporting effects of bisphosphonates in OA are summarized in Table 2.

**Table 2:** Studies reporting effects of bisphosphonates in OA

Author’s name	Year	Type	Drug/therapy	Effects in OA
She G et al. [21]	2016	Animal	Zoledronic acid	Increased the subchondral bone density, improved the microstructure, and reduced the degeneration of articular cartilage
Eriksen EF et al. [22]	2020	Review	Zoledronic acid	Reduced the articular cartilage’s deterioration
Fu SH et al. [23]	2017	Human	Bisphosphonate	Significantly reduced risk of TKA
Hayes KN et al. [24]	2020	Human	Bisphosphonate	May be protective against radiographic progress in patients with early-stage OA
Kawai T et al. [25]	2022	Human	Bisphosphonate	Reduced the rate of joint space narrowing in hips without arthritis
Fernández-Martín S et al. [26]	2021	Review	Bisphosphonate	Reduce osteoarthritic changes
Davis AJ et al. [27]	2013	Review	Bisphosphonate	There was limited evidence that bisphosphonates are effective in the treatment of OA pain
Vaysbrot EE et al. [28]	2017	Review	Bisphosphonate	Neither provides symptomatic relief nor defer radiographic progression in knee OA, but may be useful in subgroups with a high subchondral bone turnover rate

Abbreviations: *OA*, osteoarthritis; *TKA*, total knee arthroplasty

### Parathyroid Hormone (PTH)

PTH is a peptide hormone secreted by parathyroid gland and is associated with the regulation of serum calcium concentration; thus, it affects the remodeling of bone [29, 30••]. Thus, treatment with PTH analogs have the potential to treat osteoporosis and/or OA [29, 30••].

An early study of the use of PTH to treat osteoporosis showed that when given intermittently, bone formation exceeded bone resorption in the first 6–18 months of the treatment [29].

The PTH analog teriparatide (PTH (1–34)) has been studied extensively in in vitro experiments and animal models. A recent systematic review identified 22 in vivo studies for which the overall conclusions were that PTH (1–34) slowed the progression of OA by alleviating cartilage degeneration and aberrant modeling of subchondral bone and had analgesic and anti-inflammatory effects [30••]. Eleven in vitro studies were included in the review which concluded that PTH (1–34) increased the proliferation and matrix synthesis of chondrocytes. The authors of the review concluded that PTH (1–34) has potential for the treatment of OA in humans.

Our review identified 5 in vivo studies and 2 Mendelian randomization studies relevant to this article.

#### In Vivo* Studies*

Published in 2017, Zhang et al. [31] used a murine model to study the relevance of rapamycin complex 1 (mTORC1) in the initiation of OA. Prior to the study, it was known that disruption of the mTORC1 target promotes chondrocyte autophagy and survival, and thus decreases the severity of OA (experimental). The authors reported that mTORC1 activation downregulates PTH and PTH receptors in articular chondrocytes resulting in initiation of OA. Another studied published in 2017 by Ma et al. [32] used a rat model to study the effect of PTH (1–34) on cartilage degeneration. Overall, the results showed that administration of PTH (1–34) reduced histopathologic Mankin scores in the anterior cruciate ligament and medial meniscectomy-induced OA rat model. Subsequent studies have shown that PTH attenuates OA pain by remodeling subchondral bone in mice [33], that intra-articular PTH improves articular cartilage quality and a guinea pig knee OA model [34], and that PTH (1–34) attenuates cartilage degradation in a rat patellofemoral joint OA model [35].

#### Mendelian Randomization Studies

Mendelian randomization is a method of using measured variations in genes of known function to examine the causal effect of a modifiable exposure. Two Mendelian randomization studies were identified in our review, and both found a causative relation between decreased circulating PTH level and increased risk of hip and knee OA [36, 37].

### Denosumab

Denosumab is a human monoclonal antibody that targets the key bone resorption mediator RANKL [38, 39•]. Denosumab inhibits RANKL (receptor activator of nuclear factor kappa-Β ligand), and thus decreases the development of osteoclasts, which are cells that break down bone [38, 39•]. The drug is approved for the treatment of a number of conditions including osteoporosis, metastases to bone, and certain types of breast cancer [38, 39•].

Studies specifically centered on denosumab’s role in OA treatment were limited until a recent trial honed in on its application specifically for managing erosive hand OA[40]; however, some studies suggest that it can affect bone and cartilage positively and negatively. An analysis of secondary outcomes of an RCT found that administration of denosumab 1–3 days after surgery and 6 months after surgery prevented acetabular bone loss around an uncemented cup placed during total hip arthroplasty [41].

## Conclusions

Based on the evidence in the current medical literature, medications for used for the treatment of osteoporosis can also be effective for the treatment of OA. Of current medications, estrogen, SERMs, and bisphosphonates are the most studied and have the most promise as effective treatments for OA. Less is known regarding the effectiveness of PTH and denosumab, and more research is needed to determine their usefulness in the treatment of OA.

## Data Availability

All the data have been included in this published article.
